# Fighting a Losing Battle: Vigorous Immune Response Countered by Pathogen Suppression of Host Defenses in the Chytridiomycosis-Susceptible Frog *Atelopus zeteki*

**DOI:** 10.1534/g3.114.010744

**Published:** 2014-05-19

**Authors:** Amy R. Ellison, Anna E. Savage, Grace V. DiRenzo, Penny Langhammer, Karen R. Lips, Kelly R. Zamudio

**Affiliations:** * Department of Ecology and Evolutionary Biology, Cornell University, Ithaca, New York 14853; † Center for Conservation and Evolutionary Genetics, Smithsonian Institution, Washington, DC 20013; ‡ Department of Biology, University of Maryland, College Park, Maryland 20742; § School of Life Sciences, Arizona State University, Tempe, Arizona 85287

**Keywords:** *Batrachochytrium dendrobatidis*, immunogenomics, *Atelopus zeteki*, acquired immunity, immunosuppression, genetics of immunity, innate immunity, complex genetics, tolerance, complex immunity, infection, resistance

## Abstract

The emergence of the disease chytridiomycosis caused by the chytrid fungus *Batrachochytrium dendrobatidis* (*Bd*) has been implicated in dramatic global amphibian declines. Although many species have undergone catastrophic declines and/or extinctions, others appear to be unaffected or persist at reduced frequencies after *Bd* outbreaks. The reasons behind this variance in disease outcomes are poorly understood: differences in host immune responses have been proposed, yet previous studies suggest a lack of robust immune responses to *Bd* in susceptible species. Here, we sequenced transcriptomes from clutch-mates of a highly susceptible amphibian, *Atelopus zeteki*, with different infection histories. We found significant changes in expression of numerous genes involved in innate and inflammatory responses in infected frogs despite high susceptibility to chytridiomycosis. We show evidence of acquired immune responses generated against *Bd*, including increased expression of immunoglobulins and major histocompatibility complex genes. In addition, fungal-killing genes had significantly greater expression in frogs previously exposed to *Bd* compared with *Bd*-naïve frogs, including chitinase and serine-type proteases. However, our results appear to confirm recent *in vitro* evidence of immune suppression by *Bd*, demonstrated by decreased expression of lymphocyte genes in the spleen of infected compared with control frogs. We propose susceptibility to chytridiomycosis is not due to lack of *Bd*-specific immune responses but instead is caused by failure of those responses to be effective. Ineffective immune pathway activation and timing of antibody production are discussed as potential mechanisms. However, in light of our findings, suppression of key immune responses by *Bd* is likely an important factor in the lethality of this fungus.

Emerging infectious diseases arising from wildlife are rapidly gaining public and media attention as significant global threats to species diversity, ecosystem function, and human health (Chomel *et al.* 2007; Daszak *et al.* 2000; Fisher *et al.* 2012; Jones *et al.* 2008). Emerging diseases can have dire consequences for wildlife populations themselves and are now recognized as important drivers of species declines and extinctions (Smith *et al.* 2009). An emerging pathogen’s ability to “jump” between or infect multiple host species may be key to their rapid spread in natural populations (Altizer *et al.* 2011; Dobson 2004), but it is the variance in host-specific immune capabilities to cope with infection that leads to pathogen persistence in populations, as some hosts act as reservoirs for the disease in multihost communities (Cronin *et al.* 2010; Haydon *et al.* 2002). Therefore, evaluating variation in the function and efficacy of host immune responses to emerging pathogens is essential for understanding how new diseases may spread and establish in both animal and human populations.

Amphibians are experiencing global declines of unprecedented proportions; up to 50% of all species are currently at risk, making them the world’s most threatened class of vertebrates (Fisher *et al.* 2009b). The emergence of the fungal pathogen *Batrachochytrium dendrobatidis* (*Bd*) is one of the main forces driving these enigmatic declines (Lips 1999; Lips *et al.* 2006; Skerratt *et al.* 2007), especially in tropical highlands, where amphibian species diversity is greatest (Duellman 1999). *Bd* colonizes host skin and causes the disease chytridiomycosis, signs of which include lethargy, lack of appetite, cutaneous erythema, irregular skin sloughing, abnormal posture, loss of righting reflex, and eventually death in many species (Berger *et al.* 1998; Longcore *et al.* 1999; Voyles *et al.* 2011). Nonetheless, although many species have undergone catastrophic declines and/or extinctions upon arrival of *Bd*, some species appear to be unaffected or persist at reduced frequencies after chytridiomycosis outbreaks (Brem and Lips 2008; Briggs *et al.* 2010; Lips 1999; Woodhams and Alford 2005). The reasons behind such wide-ranging differences in disease outcomes, even in sympatric species, are poorly understood. Differences in host immune responses are one potential explanation (Rollins-Smith *et al.* 2002; Savage and Zamudio 2011; Woodhams *et al.* 2007). Anurans possess immunogenomic architecture and cellular mechanisms of innate and acquired immunity that are similar to those of mammals, birds, reptiles, and, to some extent, fishes (Du Pasquier *et al.* 1989; Ohta *et al.* 2006). However, limited knowledge of amphibian immune function has precluded a rigorous assessment of the mechanistic underpinnings of variation in disease susceptibility.

As of yet, no clear consensus exists regarding how acquired and innate immunity are involved in frog host responses to *Bd*. Empirical evidence indicates that differences in first line of immunological defense, innate immunity, such as skin antimicrobial peptides (AMPs), may contribute to variation in infection outcomes among hosts (Woodhams *et al.* 2007). In addition, we know that inflammation (Berger *et al.* 2005a) and infiltration of neutrophils and macrophages (Nichols *et al.* 2001) can occur when the pathogen enters frog skin cells. However, experimental infection challenges testing for sustained acquired immune responses of frogs to *Bd* infections have produced mixed results. Previous infection increases survival rates in some species (Murphy *et al.* 2011) but not others (Cashins *et al.* 2013). In addition, although inoculation with heat-killed *Bd* can elicit specific antibody responses (Ramsey *et al.* 2010), immunization had no effect on survival of two species tested (Rollins-Smith *et al.* 2009; Stice and Briggs 2010). Moreover, transcriptomic studies of three susceptible species purport a lack of robust immune responses (Rosenblum *et al.* 2009, 2012).

The variation in amphibian susceptibility to chytridiomycosis and the apparent lack of strong immunogenetic responses to experimental *Bd* challenges have led to the hypothesis that *Bd* can evade or suppress host immune responses (Ribas *et al.* 2009; Rollins-Smith *et al.* 2011; Rosenblum *et al.* 2008, 2009). Other fungal pathogens suppress or evade host immunity through a variety of mechanisms, including: (1) recognition avoidance by immune receptors via sequestration within host cells (Woods 2003); (2) digestion of its own antigens with metalloproteases to avoid recognition (Hung *et al.* 2005); and (3) interference with immune signaling (Brandhorst *et al.* 2004). Recent *in vitro* experiments have shown that *Bd* impairs splenic lymphocyte proliferation and induces apoptosis (Fites *et al.* 2013). However, these findings have not been demonstrated *in vivo* or in a species demonstrating *Bd* susceptibility in nature, and further studies are needed to assess whether this lymphocyte-killing mechanism contributes to host variation in susceptibility.

In this study, we exposed adults of the Panama Golden Frog (*Atelopus zeteki*, Figure 1A) with different infection histories (*Bd*-naïve and previously infected with an attenuated *Bd* strain) to *Bd* and compared their transcriptome-wide gene expression in three immunologically important tissues with that of uninfected control individuals. *A. zeteki* was chosen due to its high susceptibility to chytridiomycosis, which is largely responsible for the near-extinction of this species in the wild (Gewin 2008). This critically endangered frog, and other species within the genus *Atelopus*, have become a flagship for public awareness to the plight of amphibians in the face of the spread of *Bd* and the species is the focus of an intensive captive-breeding program (Blaustein and Dobson 2006; Gewin 2008). Our objectives were twofold: (1) characterize transcriptomic changes related to immune responses of *A. zeteki* to *Bd* and (2) ascertain whether there is functional genomic evidence of *Bd* suppression of immune functions contributing to the high susceptibility of this species.

**Figure 1:**
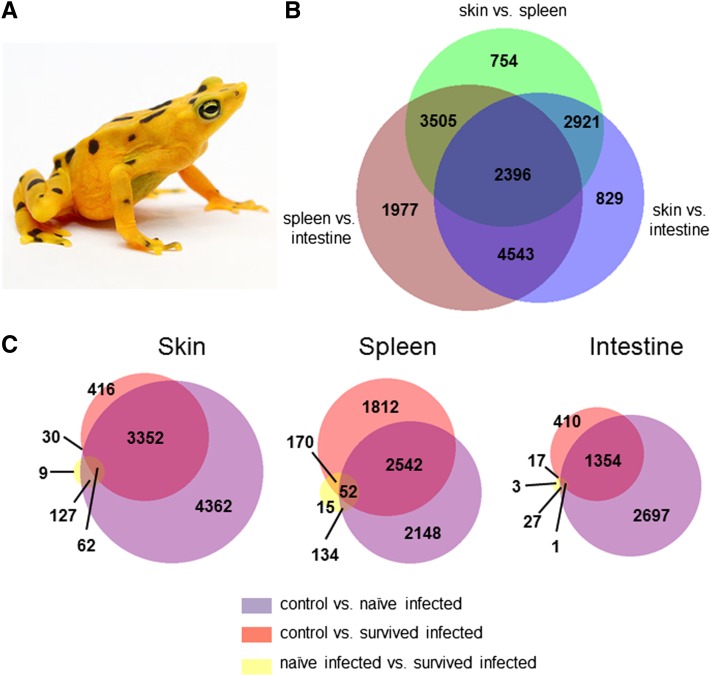
Number of significant differentially expressed genes among *Atelopus zeteki* tissues and infection groups. Area-proportional Venn diagrams summarizing the number of significantly differently expressed genes (<0.05 FDR corrected *P* value) of (A) *Atelopus zeteki*, (B) among three tissues, and (C) among treatment groups within each tissue. Photo courtesy of Brian Gratwicke.

## Materials and Methods

### Experimental infections

Ten captive-bred adult (approximately 18-mo-old) *Atelopus zeteki* from the same clutch of a full-sibling mating at the Maryland Zoo in Baltimore were used for experiments. These animals were surplus to the Golden Frog Project captive breeding program at Maryland Zoo. We chose full siblings for our experiment because genetic polymorphisms may account for a substantial proportion of variation in gene expression (Stranger *et al.* 2007) and biological variation in small sample sizes can hinder the ability to detect meaningful differences in gene expression between treatment groups (Hansen *et al.* 2011). Differential gene expression studies using related individuals also pose limitations as the transcriptomic response of one genetic lineage may not hold for the entire species. However, as this species is critically endangered and therefore availability of experimental animals is limited, we opted to reduce genetic diversity due to small available sample sizes.

All individuals had no previous exposure to *Bd*, appeared healthy, and tested negative for *Bd* at the start of the experiment. Individuals were paired by weights and placed into one of two treatment groups; control and naïve-infected. Two additional frogs from the same clutch, previously exposed to *Bd* strain JEL-427-P39 (30 mL of 1 × 10^2^ zoospores/mL) but which survived *Bd* infections (*i.e.*, developed substantial *Bd* loads yet survived with reduced infection without intervention), were assigned as a third, survived-infected, treatment group (Langhammer *et al.* 2013).

Animals in all three treatments were housed individually in sphagnum-moss filled plastic shoeboxes with a water dish, in a laboratory maintained at 21-22° with a 12:12-hr light:dark photoperiod. All housing materials were replaced every 7 d, water dishes changed every 3 d, and animals were fed three to four crickets or 10−15 fruit flies (*Drosophila melanogaster*) sprinkled with Herptivite (Rep-Cal Research Labs) every 3 d.

For experimental infections we used *Bd* strain JEL-423 cultured and cryopreserved from a field-infected *Hylomantis lemur*, during the epidemic outbreak at El Copé, Panama in 2004 (Lips *et al.* 2006). A hemocytometer was used to create a solution containing 1 × 10^3^ zoospores/mL. The infection treatment frogs (five naïve, two survived) were individually exposed to 30 mL of the *Bd* solution (30,000 zoospores) in deli containers for 10 hr. The five control individuals were exposed to a sham solution of distilled water and tryptone. Although it is unknown whether our inoculation dose reflects exposure levels in nature, earlier studies show that *Bd*-dose does not affect infection likelihood or mortality but does influence time to death (Carey *et al.* 2006). Both JEL-423 and JEL-429 strains belong to the hypervirulent global pandemic lineage of *Bd* (Farrer *et al.* 2011).

Throughout the experiment, we used a fresh pair of latex powder-free gloves when handling each individual. All individuals were assayed for *Bd* infection status and load at day 8 postinoculation, and thereafter every 3−4 d. We swabbed the abdomen, drink patch, hands, and feet five times each with a sterile cotton tipped swab, which was then stored in capped tubes containing 30 μL of 70% ethanol (Hyatt *et al.* 2007). We tested swabs for *Bd* using PrepMan Ultra and ran samples in singlicate Taqman quantitative polymerase chain reaction (qPCR) (Boyle *et al.* 2004). We ran each plate with JEL-427 standards of 0.1, 1, 10, 100, and 1000 zoospore genomic equivalents to determine *Bd* presence and infection intensity. We categorized individuals as *Bd*-positive when qPCR results showed an infection load greater than or equal to one *Bd* zoospore genomic equivalent (Kriger *et al.* 2006).

Individuals were monitored daily for clinical symptoms of chytridiomycosis, and we killed those that had lost righting abilities by applying 20% Benzocaine to the venter. All individuals were sexed, weighed, and measured at the end of the experiment. Control animals were killed on day 33, the day the last infected individual was killed. The number of *Bd* zoospores that infect an individual during inoculation is variable and can cause variations in length of time to death among individuals (Carey *et al.* 2006). Thus, we harvested tissues when all individuals were biologically experiencing the same clinical signs of chytridiomycosis, rather than choosing a fixed time point when individuals vary in disease progression. Immediately after euthanasia, frogs were dissected using sterilized instruments, and skin (drink patch), spleen, and small intestine tissue samples were harvested from each individual. These tissues were chosen as (1) skin is the primary site of infection for *Bd* (Longcore *et al.* 1999), (2) the spleen is the major lymphoid organ in frogs, involved in producing leukocytes and other immune cells (Tischendorf 1985), and (3) small intestine has been shown to possess large numbers of B cells and expresses T-cell−independent immunoglobulins in other anuran amphibians (Mußmann *et al.* 1996). Tissue samples were immediately placed in RNALater (Invitrogen) and stored at −80° prior to RNA extraction and library preparation. All experiments were performed with approval from and in accordance with the ethical standards of the US Institutional Animal Care and Use Committee under protocol R-12-98.

### Transcriptome sequencing

Total RNA was extracted from each tissue sample separately using RNAdvance tissue kit (Beckman Coulter Inc.). We quantified and assessed RNA integrity using a 2100 Bioanalyzer total RNA nano assay (Agilent Technologies). All samples had RNA integrity values greater than 8.0. Libraries were generated using the Illumina TruSeq RNA sample preparation kit v2 (low-throughput protocol) according to the manufacturer’s instructions (Illumina, San Diego, CA). Briefly, 0.4−1.2 µg of RNA was subjected to mRNA selection using poly-T oligo-attached magnetic beads followed by chemical fragmentation (6 min, 94°). The cleaved RNA fragments were then copied into first-strand cDNA using SuperScript II reverse transcriptase (Invitrogen) and Illumina proprietary random hexamer primers. After second-strand synthesis using Illumina supplied consumables, the cDNA was amplified with reagents of the same kit and ligated to barcoded adapters. The final libraries were amplified using 14 PCR cycles. We quantified and assessed library quality on a Bioanalyzer 2100 and randomly pooled equimolar samples in four lanes of the Illumina flowcell (eight or nine samples per lane).

### Transcriptome assembly and functional annotation

After Illumina standard quality control filtering, read quality for each sample was visualized using FastQC version 0.10.0 (Andrews 2010). All samples had greater than average GC content in the first 15 bp due to utilization of random hexamer primers during library preparation. Therefore we used Trimmomatic version 0.27 (Lohse *et al.* 2012) to; (1) remove the first 15 bp of each read, (2) trim any Illumina adapter sequence, (3) trim the 5′ and/or 3′ end of reads where quality score dropped below Q20, (4) trim anywhere within each read where a 5-bp window drops below Q20, and (5) discard any trimmed reads less than 36 bp long. This ensured only the highest quality reads were used for subsequent *de novo* assembly.

Reads from all individuals and tissues were pooled to assemble a consensus transcriptome. To overcome the challenges associated with high memory and computing requirements necessary for *de novo* assembly of large numbers of sequence reads, we performed Trinity (Grabherr *et al.* 2011) *in silico* read normalization procedure before assembly. Assembly was performed using Trinity’s default parameter settings on a high-performance cluster with 64 CPUs and 512GB RAM. We filtered out transcripts with expression support of less than five reads per million mappable reads in at least two samples to eliminate low-level expression noise (Harrison *et al.* 2012; Moghadam *et al.* 2013).

Blast2GO version 2.5.0 (www.blast2go.com) was used to functionally annotate the assembled transcriptome. First, the longest sequence of each Trinity component (roughly equivalent to a single gene) was extracted from the assembly using custom Perl scripts. Next, these sequences (hereafter contigs) were aligned via Blastx to the National Center for Biotechnology Information (NCBI) nonredundant protein database, retaining up to 20 hits with a minimum E-value of 1 × 10^−6^ and minimum bit score of 55. Any contig aligning to the *Bd* transcriptome (*Batrachochytrium dendrobatidis* Sequencing Project, Broad Institute of Harvard and MIT; www.broadinstitute.org) was removed from further analyses. Gene ontology (GO; www.geneontology.org) mapping was performed by extracting the GO terms associated with homologies identified by Blastx and producing a list of GO annotations represented as hierarchical categories of increasing specificity. We retained annotations with a minimum E-value of 1 × 10^−06^, a minimum annotation cut-off of 55, and a GO weight of 5. GO annotations were enhanced using the annotation augmentation tool ANNEX (Myhre *et al.* 2006). Finally, we performed InterPro (Quevillon *et al.* 2005) searches remotely from BLAST2GO via the InterPro EBI web server and merged InterProScan GOs with the original GO annotations.

### Gene expression analysis

We analyzed differential gene expression (DE) using the edgeR (Robinson *et al.* 2010) R package (R version 2.15.2, R Development Core Team). Analysis in edgeR consisted of (1) only including contigs with more than five counts per million from at least two individuals; (2) estimating tagwise dispersion and normalization factors; and then (3) DE was tested using the exact test and the false-discovery rate (FDR) corrected *P*-value of less than 0.05 was considered to be evidence of DE. PowerAtlas (Page *et al.* 2006) analysis of raw *P*-values showed that comparisons using the smallest treatment group (survived-infected, n = 2) would correctly identify ∼85% of truly differentially expressed genes and ∼76% of truly nonsignificant genes. Thus, after correction for multiple testing, our lists of differentially expressed genes are highly conservative but necessary given our small sample sizes. To get a broad overview of DE patterns within each tissue type, highly differentially expressed genes (<0.001 FDR, >fourfold change) were clustered via a k-means algorithm and plotted on heatmaps using Trinity-provided and custom Perl and R scripts. GO functional enrichment tests (with FDR correction for multiple tests) were carried out on each cluster via Blast2GO to detect significantly overrepresented biological processes, molecular functions, and cellular components.

## Results

### Experimental infections

Experimental animals consisted of 1 male (control group) and 11 females. Despite the potential for sex differences in gene expression levels, subsequent analyses including and excluding the male control animal were not substantially different from one another. Overall expression patterns and specific genes found to be significantly different between control and infected groups remained consistent, albeit with minor differences in fold-change and significance values. Therefore, we included all individuals, regardless of sex, in our analyses. Weight and snout-to-vent length of the treatment groups did not differ significantly at the start of the experiment (weight; F_2,8_ = 0.222, *P* = 0.806, length; F_2,8_ = 0.030 *P* = 0.971). All of the five control animals remained *Bd*-negative for the duration of the experiment. All infected animals (five naïve-infected, two survived-infected) were positive for *Bd* zoospores 8 d postinoculation (mean zoospores = 34,015, S.E. = 15,741) and for the remainder of the experiment. One naïve-infected individual died before being killed; therefore, that individual was removed from the study. The remaining six infected frogs lost righting reflexes and were killed between 22 and 33 days postinoculation. Mean zoospore load at death for experimentally infected animals was 6,819,500 (S.E. = 2,431,929). No significant differences in survival time (t = 0.594, *P* = 0.585) or infection intensities (t = 1.896, *P* = 0.106) were found between naïve-infected and survived-infected animals. However, with only two individuals in the clear-infected category, we cannot completely rule out the possibility that previous exposure affects survival time or infection load in the species.

### Transcriptome assembly and annotation

Skin, spleen, and small intestine tissue samples from 11 *Atelopus zeteki* (five uninfected control, four naïve infected, and two survived infected) were sequenced on four lanes of Illumina HiSeq, resulting in more than 896 million 100 bp single-end reads in total. Sequences are deposited in the NCBI Short Read Archive under the submission accession number SRP029154. The average number of raw reads per sample was approximately 27.2 million, reduced to 25.9 million after quality filtering and trimming. Total number of filtered reads, pooled from all samples and after normalization, was approximately 66.5 million. These were assembled using Trinity, and our *de novo* assembly of the transcriptome yielded 447,764 transcripts, with a mean length of 786 bp and N50 length of 1596 bp. After filtering out low expression transcripts by setting a threshold of at least five reads per million mappable reads in at least two samples, we retained 116,504 transcripts, representing 40,084 expressed gene fragments and their isoforms. We expect that the vast number of minimally expressed contigs that fall below our threshold to largely represent transcriptional errors such as intron expression, exon chimeras, and sequencing and assembly errors common to current *de novo* assembly techniques (Moghadam *et al.* 2013). Of the assembled genes, 15,252 (38.1%) had at least one significant hit against the nonredundant NCBI protein database, and of these, 12,056 (79.0%) were successfully annotated with GO terms. The vast majority—just over 72%—of best hits were to *Xenopus* species.

### Broad-scale patterns of gene expression

As expected, a significant proportion of genes were differentially expressed among the three tissue types, regardless of infection status (Figure 1B). Within each tissue type, we identified a large number of genes differentially expressed between the control (uninfected) frogs and both infected groups (Figure 1C). However, far fewer genes were differentially expressed between naïve-infected and survived-infected individuals (Figure 1C).

**Figure 2:**
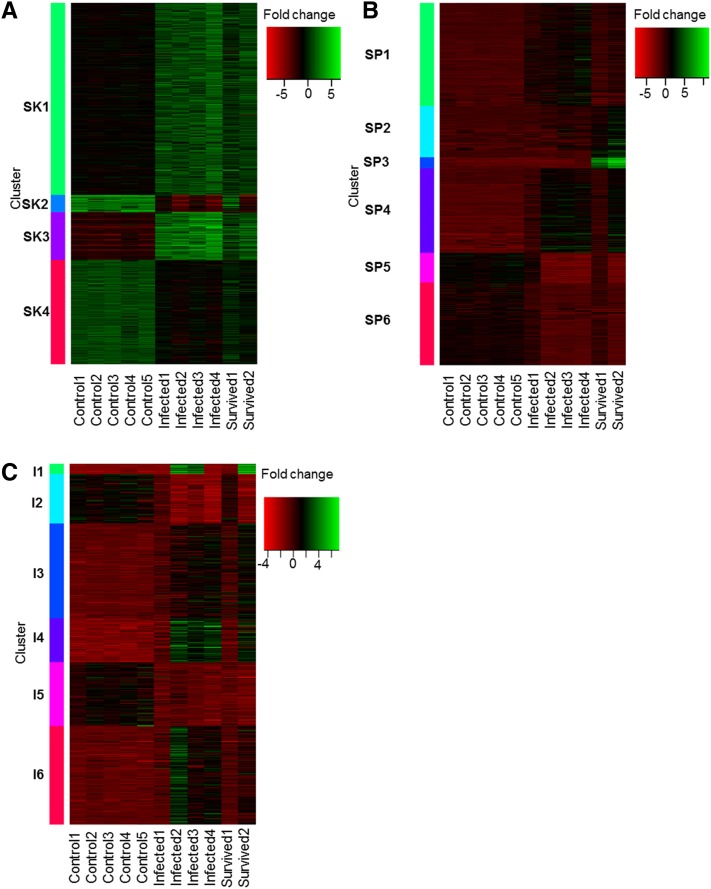
Heatmaps summarizing differential gene expression in *Atelopus zeteki*. (A) skin, (B) spleen, and (C) small intestine, clustered (via k-means clustering algorithm) by similarity of expression. Each row represents a gene within a minimum FDR-corrected *P*-value of 0.001 and at least fourfold difference in expression between samples (columns).

Clustering of skin gene expression patterns revealed four clusters (SK1 to SK4, Figure 2A). SK1 and SK3 could be broadly characterized as increased expression in infected frogs (more so in the naïve group) and both had an overrepresentation of immune system functions (Table 1). These included terms such as “response to fungus” (GO:0009620), “inflammatory response” (GO:0006954), and “humoral immune response mediated by circulating immunoglobulin” (GO:0002455). The two other skin clusters (SK2 and SK4) were significantly enriched for terms such as “water transport” (GO:0006833), “keratinocyte differentiation” (GO:0030216), and “collagen fibril organization” (GO:0030199) and generally exhibited decreased expression in the infected groups.

**Table 1:** Summary of gene cluster expression patterns and overrepresented GO terms

Cluster*^a^* ID	Expression Pattern*^b^*			No. of Genes (No. Annotated)	No. of Overrepresented GO Terms	Examples of Overrepresented GO Terms*^c^*
	Control*^d^*	Naïve*^e^*	Survived*^f^*			
Skin						
SK1	−1.1	+1.1	+0.5	1395 (654)	364	Response to fungus, humoral immune response mediated by circulating immunoglobulin, B-cell activation involved in immune response, cytokine secretion, leukocyte migration
SK2	+2.4	−2.7	−0.7	129 (55)	13	Water transport, keratinocyte differentiation, cellular response to metal ion
SK3	+2.3	−2.3	−1.1	349 (172)	20	Inflammatory response, immune response, defense response to bacterium, response to Lipopolysaccharide, response to cytokine stimulus
SK4	+1.1	−1.0	−0.7	755 (475)	58	Epithelial cell proliferation, collagen fibril organization, electron transport, DNA replication, heme
						Catabolic process
Spleen						
SP1	−0.8	+1.1	−0.1	373 (222)	8	Immune response, response to chemical stimulus
SP2	−0.6	−0.2	+1.8	187 (104)	0	No significant enrichment
SP3	−1.5	−1.5	+6.9	42 (39)	19	Proteolysis, serine-type endopeptidase activity, metallocarboxypeptidase activity, hydrolase activity
SP4	−1.3	+1.2	+0.9	308 (168)	21	Cell communication, response to hypoxia, circulatory system development, signaling
SP5	+1.6	−1.5	−1.2	108 (55)	64	Oxygen transport, immune system development, cellular ion homeostasis, Gamma-delta T-cell activation, gamma-delta T-cell differentiation
SP6	+1.0	−0.8	−0.8	301 (166)	16	Alpha-beta T-cell differentiation, cytokine production, regulation of cell activation, hemopoiesis
Intestine						
I1	−1.8	+1.2	+2.2	23 (23)	23	Serine-type peptidase activity, proteolysis, collagen catabolic process, metalloexopeptidase activity
I2	+1.2	−1.2	−0.5	117 (76)	21	Oxidation-reduction process, lipid metabolic process, monosaccharide metabolic process
I3	+0.8	−0.6	−0.7	225 (93)	0	No significant enrichment
I4	−1.5	+1.6	+0.5	103 (53)	0	No significant enrichment
I5	+0.9	−0.7	−0.9	152 (107)	57	Mitotic cell cycle, protein folding in endoplasmic reticulum, DNA replication, regulation of nuclease activity, chromosome organization
I6	−0.8	+1.0	0.0	234 (166)	58	Immune system development, programmed cell death, regulation of response to stress, chronic inflammatory response, signal transduction

a Clusters defined by k-means clustering.

b Average log_2_-fold change.

c Full list of overrepresented GO terms are provided in Table S1.

d Control uninfected.

e *Bd*-naïve infected.

f Previously survived *Bd* infected.

Spleen gene expression clusters totaled six (SP1−SP6, Figure 2B) and all but one (SP2) possessed significantly overrepresented GO terms (Table 1). Of note, SP5 and SP6 indicated decreased expression of immune system activation terms in both naïve- and survived-infected frogs, specifically in both alpha-beta and gamma-delta T-cell activation and differentiation (GO:0046629, GO:0042492, GO:0046631, GO:0046632). In addition, SP3, a small but extremely highly expressed set of genes in the survived-infected frogs were enriched for “serine-type endopeptidase activity” (GO:0004252), “metallocarboxypeptidase activity” (GO:0004181), and “hydrolase activity” (GO:0016787). The SP3 gene cluster also included massively increased expression of protease inhibitors (*e.g.*, serpin I2, log_2_-fold-change = 12.86, *P* = 2.04 E^-100^) in survived-infected frogs.

Expression clustering of intestine tissues revealed six clusters (I1−I6, Figure 2C), of which four were significantly enriched for GO terms (I1, I2, I5, I6; Table 1). One cluster exhibiting substantial increases in expression in infected groups (I1), similar to SP3, contained serine-type endopeptidase (GO:0004252) and metalloexopeptidase (GO:0008235) activity terms. In addition, I6 also showed minor increases in expression, with overrepresented terms including “immune system development” (GO:0002520) and “chronic inflammatory response” (GO:0002544). Clusters I2 and I5 showed decreased expression in infected individuals and were enriched for key metabolic processes such as lipid and monosaccharide metabolism plus cell-cycle components such as DNA replication and mitosis. Decreased expression of genes related to metabolic processes is likely a reflection of reduced food intake, common during *Bd* infections. However, in contrast to skin and spleen samples, cluster expression differences between treatment groups were less defined. For example, individuals infected-1 and survived-1 showed expression patterns more similar to controls than the other infected animals in the majority of the intestine clusters. The full list of significantly enriched GO terms and their *P*-values is provided in Supporting Information, Table S1.

### Immune responses in *Bd*-infected frogs

We found 371 genes to be differentially expressed between infected and control frogs that are identified as immunogenetic (Table S2, Table S3, and Table S4). However, given the relative paucity of amphibian immunological data, many genes discussed herein have not yet been fully characterized in amphibians. It should be noted that, given the stringent annotation criteria used, we are referring to the most homologous genes and their functions in other vertebrates. Within the great number of differentially expressed immune genes found, the largest group could be broadly classified as encoding cytokine signaling molecules and their receptors. These included increased expression of all major groups of cytokines that are part of the vertebrate immune system; interferons, interleukins, tumor necrosis factors, and chemokines (Table 2).

**Table 2:** Number of differentially expressed immune-related genes and percentage with expression increases in three tissues of *Atelopus zeteki*

Category (Tissue)	Naïve *vs.* Control		Survived *vs.* Control		Naïve *vs.* Survived	
	No.	% up	No.	% up	No.	% up
B cells						
Skin	16	75.0	5	80.0	1	100.0
Spleen	18	38.9	15	40.0	1	100.0
Intestine	6	83.3	3	100.0	1	100.0
Complement						
Skin	16	81.3	13	92.3	0	−
Spleen	16	100.0	7	100.0	2	50.0
Intestine	6	100.0	4	25.0	0	−
Immunoglobulins						
Skin	8	75.0	9	88.9	4	0.0
Spleen	20	80.0	9	77.8	3	100.0
Intestine	3	100.0	1	100.0	0	−
Interferons						
Skin	27	96.3	17	100.0	0	−
Spleen	17	35.3	22	18.2	0	−
Intestine	10	100.0	4	100.0	0	−
Interleukins						
Skin	36	91.7	19	94.7	8	87.5
Spleen	24	70.8	11	36.4	4	100.0
Intestine	11	100.0	4	100.0	0	−
Macrophages						
Skin	8	75.0	6	83.3	1	100.0
Spleen	10	80.0	5	40.0	2	100.0
Intestine	6	66.7	0	−	0	−
Major and minor histocompatibility						
Skin	3	100.0	6	100.0	0	−
Spleen	3	33.3	4	75.0	0	−
Intestine	0	−	0	−	0	−
Nuclear factor-κβ						
Skin	6	100.0	4	100.0	0	−
Spleen	4	100.0	2	100.0	0	−
Intestine	2	100.0	1	0.0	0	−
Toll-like receptors						
Skin	6	66.7	2	100.0	0	−
Spleen	6	100.0	3	33.3	1	100.0
Intestine	3	66.7	0	−	1	100.0
Tumor necrosis factors						
Skin	28	92.9	17	100.0	3	100.0
Spleen	20	80.0	15	66.7	1	100.0
Intestine	16	87.5	7	71.4	0	−
T cells						
Skin	8	75.0	3	33.3	0	−
Spleen	35	17.1	30	10.0	0	−
Intestine	4	50.0	1	0.0	0	−
Other cytokines						
Skin	16	93.8	9	77.8	1	100.0
Spleen	18	72.2	12	41.7	4	75.0
Intestine	6	100.0	3	100.0	0	−
Other immune-related genes						
Skin	22	90.9	16	87.5	3	66.7
Spleen	19	63.2	18	44.4	7	57.1
Intestine	12	75.0	6	100.0	2	100.0

A complete list of genes, fold-changes, and significance is available in Table S2, Table S3, and Table S4. Control, uninfected control; naïve, *Bd*-naïve infected; survived, previously survived *Bd* infected.

Genes encoding interleukins exhibited some of the greatest increases in expression in infected animals, particularly in the skin. Interleukin (IL)-10 and IL-17 both increased expression by greater than 4000-fold in the skin of naïve-infected frog but was not significantly different between survived-infected and control animals (Table S2). Significant increases in IL-17 levels also were found in the spleen of the naïve-infected but not in the survived-infected group (Table S3). Inflammatory interleukins IL-1, -6, -8, -12, and -18 also were found to have significant increased expression in Bd-naïve frogs (Table S2). However, all interleukins had either lower or nonsignificant increases in expression in frogs previously exposed to *Bd*.

Many immune-related chemokines and their receptors showed increased expression in skin, spleen, and intestine tissues of infected frogs, including CCL4, CCL19, CCL27 CXCL10, CXCL14, CCR1, CCR3, and CCR4 (Table 2). A number of components of the classical complement system (*e.g.*, C1q, C5) showed increased expression in all three tissues studied (Table S2, Table S3, and Table S4), with the greatest increases seen in the skin of naïve-infected frogs. Increased expression was also found in the skin of survived-infected individuals, but to a lesser extent in the spleen and intestine. Alternative pathway expression (*e.g.*, complement factors b, d, h) in the spleen of infected animals is also increased, but inconsistent patterns were observed in the skin and lower expression levels than uninfected in the intestine. Infected *A. zeteki* exhibited increased expression in seven TLR genes in at least one of the three tissues studied (Table 2). TLR2 had moderately increased expression in all tissues of the naïve-infected group (Table S2, Table S3, and Table S4). TLR5 had the greatest increase in expression in all tissues, particularly in the skin of naïve-infected frogs (Table S2).

**Figure 3:**
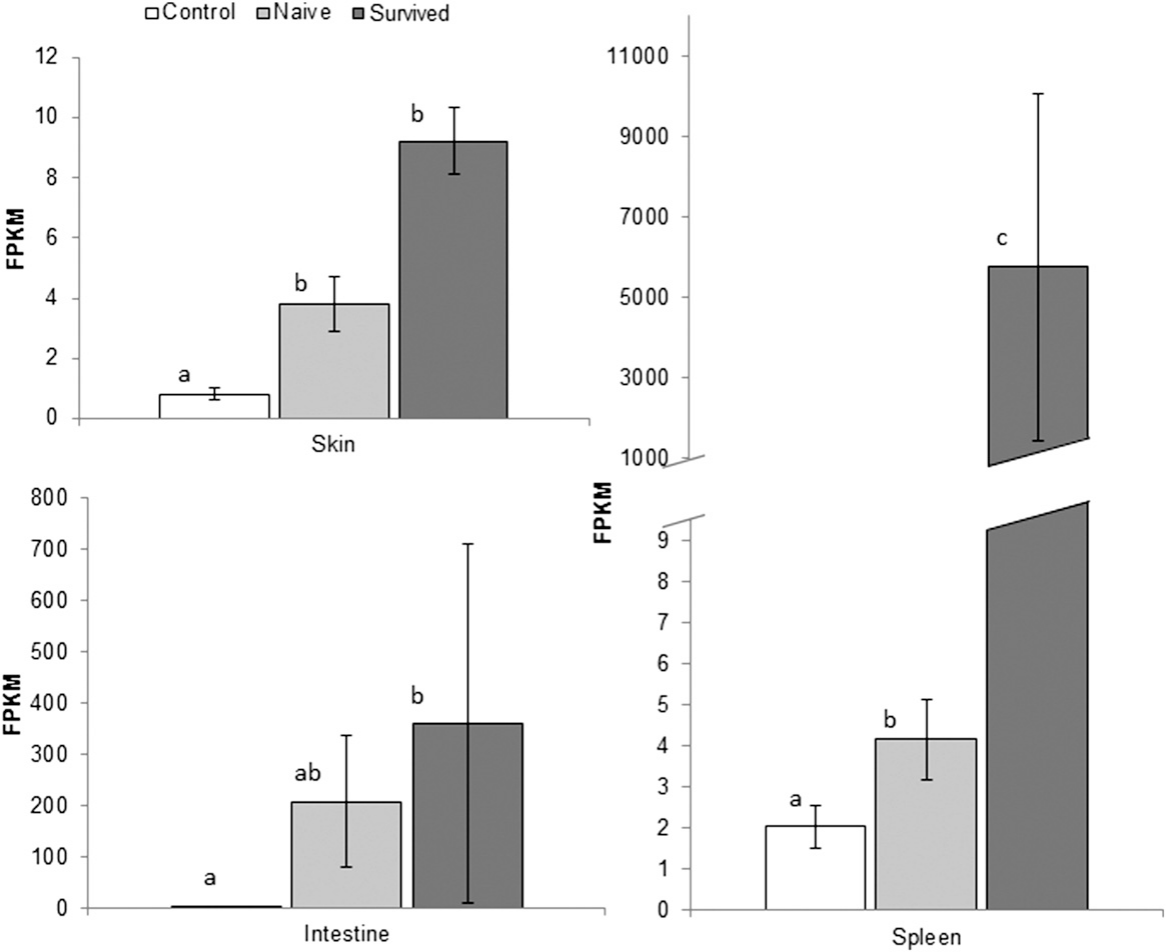
Differential gene expression of chitinase 1 in *Atelopus zeteki*. Results of exact tests for expression differences measured by fragments per kilobases per million mapped (FPKM), in chitinase 1 gene in three tissues of *Atelopus zeteki*. Letters indicate significant differences among treatment groups (no significant difference among groups with same letter, FDR corrected *P*-value <0.05) and error bars represent ± 1 SE.

The gene encoding the chitinase CHIT1 was found to have significant increases in expression in skin and spleen tissues in both naïve-infected and survived-infected groups compared with the controls (Figure 3). Expression increases were significantly greater in the spleen of survived-infected frogs compared with naïve-infected (with a similar but nonsignificant trend in the skin). CHIT1 exhibited one of the greatest expression changes of all genes in the spleen of survived-infected individuals (greater than 99.6% of genes, Table S3).

Genes encoding receptors and other proteins specific to B cells were found to have increased expression in both infected groups within the skin and intestine, for example FC receptor 5-like (Table 2, Table S2, and Table S4). Components of T-cell receptors (*e.g.*, T-cell receptor beta) and T-cell−specific CD markers such as CD4 and CD3 also were found to have elevated expression in the skin of infected animals (Table 2 and Table S2). In addition, both class I and II MHC genes showed significant increases in expression in both naïve-infected and survived-infected skin samples compared with uninfected controls (Table 2 and Table S2). However, we found lower expression levels in a number of B-cell−related genes, such as “B-cell lymphoma leukemia 11b isoform 2,” in the spleen of both infected groups. Markers specific to T cells such as CD4 and CD8 also were significantly lower in both naïve-infected and survived-infected spleens (Table 3). In addition, a large number of T-cell receptor components were also decreased in the spleen of infected individuals including alpha, beta, gamma, and delta chains (Table 3).

**Table 3:** T -cell receptor gene expression differences

Contig ID	Description	Log_2_-Fold Change *(FDR P-Value)*											
		Naïve Infected						Survived Infected					
		Skin		Spleen		Intestine		Skin		Spleen		Intestine	
comp185333_c1	T-cell receptor alpha chain			**−2.69**	*2.14 E-10*	**−1.44**	*4.75 E-3*			**−2.07**	*2.14 E-5*		
comp100948_c0	T-cell receptor alpha chain			**−4.20**	*4.44 E-8*					**−3.29**	*2.15 E-6*		
comp136812_c0	T-cell receptor alpha chain			**−3.07**	*1.71 E-7*					**−2.86**	*9.98 E-5*		
comp183357_c0	T-cell receptor alpha chain v region			**−2.73**	*1.48 E-15*					**−1.96**	*1.15 E-4*		
comp186997_c2	T-cell receptor beta	+3.21	*1.80 E-7*	**−2.76**	*3.79 E-14*					**−1.61**	*1.93 E-3*		
comp193946_c0	T-cell receptor beta variable 19			**−2.96**	*5.05 E-8*					**−2.34**	*9.66 E-5*		
comp153538_c0	T-cell receptor beta chain			**−3.41**	*3.89 E-11*					**−2.43**	*6.85 E-5*		
comp151427_c0	T-cell receptor beta chain			**−3.24**	*4.78 E-10*					**−1.75**	*1.52 E-3*		
comp137067_c0	T-cell receptor beta chain			**−3.24**	*4.68 E-12*					**−2.27**	*4.92 E-5*		
comp167921_c0	T-cell receptor beta chain			**−2.94**	*2.87 E-10*					**−1.98**	*1.37 E-3*		
comp153207_c0	T-cell receptor beta chain			**−2.90**	*2.12 E-7*								
comp164781_c0	T-cell receptor beta chain			**−2.89**	*1.47 E-12*					**−2.04**	*1.23 E-3*		
comp100980_c0	T-cell receptor beta chain			**−2.86**	*2.99 E-8*					**−1.79**	*4.20 E-3*		
comp148731_c0	T-cell receptor beta chain			**−2.69**	*3.49 E-6*					**−2.10**	*1.54 E-3*		
comp172614_c0	T-cell receptor delta chain			+2.00	*4.30 E-6*								
comp180632_c0	T-cell receptor delta chain			**−2.24**	*4.79 E-10*					**−1.00**	*1.03 E-2*		
comp176041_c0	T-cell receptor gamma chain			**−3.74**	*8.57 E-12*					**−4.27**	*2.28 E-10*		
comp178628_c0	T-cell receptor gamma chain			**−1.82**	*5.20 E-4*					**−1.63**	*5.76 E-3*		
comp159481_c0	CD3e epsilon (CD3-TCR complex)	+3.57	*4.96 E-10*	**−1.78**	*1.68 E-7*					**−1.52**	*4.46 E-4*		
comp185118_c0	CD4 precursor	+2.00	*8.73 E-5*	**−2.1**	*2.61 E-8*			+1.51	*0.001*	**−1.50**	*2.51 E-3*		
comp171746_c0	CD8 alpha			**−2.89**	*1.67 E-8*					**−1.87**	*6.89 E-4*		
comp180074_c0	CD8 beta					+0.87	*4.55 E-2*						

Comparisons between uninfected (control) and infected (naïve *vs.* control and survived *vs.* control) *Atelopus zeteki*. *P* values after FDR correction for multiple tests. Significant decreases in expression are highlighted in bold. FDR, false-discovery rate.

**Figure 4:**
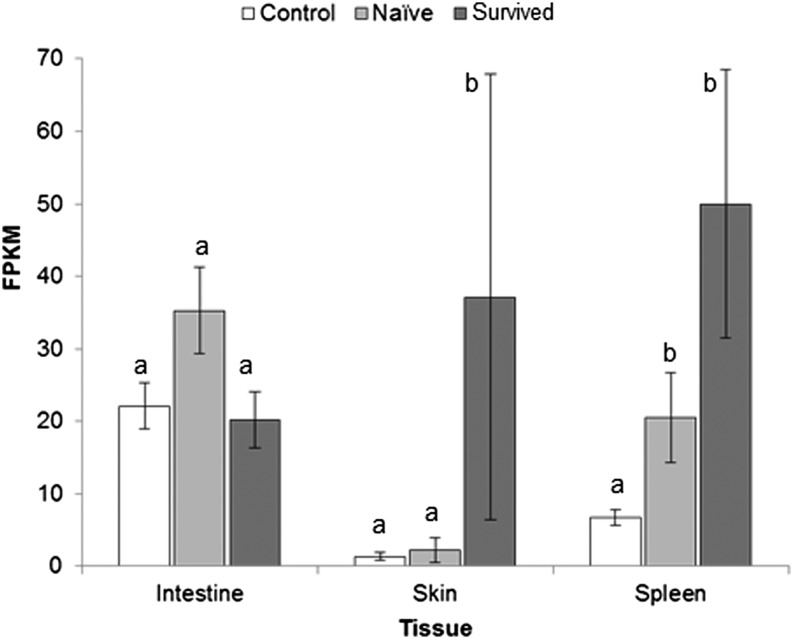
Differential gene expression of immunoglobulin heavy chain constant region in *Atelopus zeteki*. Results of exact tests for expression differences, measured by fragments per kilobases per million mapped (FPKM) in immunoglobulin heavy chain constant region in three tissues of *Atelopus zeteki*. Letters indicate significant differences among treatment groups (no significant difference among groups with same letter, FDR corrected *P* value <0.05) and error bars represent ± 1 SE.

Increased immunoglobulin expression was observed in all three tissues of infected frogs. Although only a few contigs identified as immunoglobulin-related had increased expression in intestine tissues, 16 were elevated in the spleen of naïve-infected frogs, many of which were also found in survived-infected frogs (Table 2, Table S2, and Table S4). In the skin, a substantial number were elevated in survived-infected compared with controls but not in naïve-infected animals (Table S3). For example “immunoglobulin heavy chain constant region” had greater expression levels in both infected groups compared with controls in the spleen. But in the skin, this gene had similar expression levels in uninfected and naïve-infected frogs yet significantly increased expression in survived-infected skin samples (Figure 4).

## Discussion

### Broad-scale gene expression profiles of frogs infected with *Bd*

Chytridiomycosis is one of the main drivers of the recent dramatic declines in amphibian populations worldwide (Lips 1999; Lips *et al.* 2006; Skerratt *et al.* 2007). The efficacy of species immune responses to *Batrachochytrium dendrobatidis*—the causative agent of the disease—is proposed to be a key factor in determining disease outcome (Rollins-Smith *et al.* 2002; Savage and Zamudio 2011; Woodhams *et al.* 2007). High-throughput methods of gene expression profiling, such as RNA-Seq, provide the opportunity for in-depth characterization of host responses and to elucidate important mechanisms in the dynamics of this disease. Our transcriptome-wide study of a highly susceptible species, *Atelopus zeteki*, identified a large number of genes differentially expressed between the control (uninfected) frogs and both infected groups. However, far fewer genes were differentially expressed between naïve-infected and survived-infected individuals. This finding suggests that broad-scale gene expression changes are similar in frogs challenged by *Bd* regardless of their previous infection history, and only a small subset of genes are differently expressed among naïve and previously infected individuals. Of the three tissues studied, the spleen, a major lymphoid organ of frogs, showed the greatest dissimilarity in expression differences between naïve and survived-infected frogs.

Although these results could be interpreted as indicative of significant changes in immunogenetic responses to infection due to prior exposure, comparisons between *Bd*-naïve and *Bd*-survived groups should be interpreted with degree of caution, given the small sample size of the survived-infected group (n = 2). Small sample size for individuals naturally surviving infection (*i.e.*, without interventions such as fungicidal treatment) was unavoidable for this study due to this species’ high-susceptibility to *Bd*; only two of 60 frogs infected with *Bd* survived the original experiment (Langhammer *et al.* 2013). Despite the fact that frogs from both studies were highly inbred siblings, we cannot infer from our data whether the transcriptomic responses of the two surviving individuals are a result of innate genetic predisposition or due to their previous exposure to a form of the fungal pathogen. In addition, though reducing genetic variability by utilizing clutch-mates can be beneficial for small sample sizes (see *Materials and Methods*), it may not fully capture the expression responses of the entire species. Future infection studies should explore attenuated (Langhammer *et al.* 2013) or naturally less virulent (Berger *et al.* 2005b; Fisher *et al.* 2009a) *Bd* strains and in more genetically diverse hosts as a means of obtaining greater sample sizes of cleared individuals and to disentangle the effect of host genetics and previous exposure on adaptive immunity.

Clustering of skin gene expression patterns revealed two sets of genes, with significantly greater expression in infected frogs, significantly enriched for many immune-related GO terms including inflammatory, interleukin, B-cell, T-cell, and immunoglobulin responses. This indicates that, in this species, there is increased expression of both innate and adaptive immune responses in skin cells, the primary site of infection for *Bd*. The remaining skin clusters, with overall decreased expression in infected individuals, included terms involved in skin structure (*e.g.*, collagen fibril organization) and water transport. These results are consistent with previous observations that *Bd* pathogenicity occurs via disruption of skin integrity and water/ion imbalance (Rosenblum *et al.* 2012; Voyles *et al.* 2009).

In the spleen, we found a small cluster of genes with large increases in expression only in frogs previously exposed to *Bd*, which was enriched for various protease GO terms and also included protease inhibitors. Proteases, particularly serine peptidases, play important roles in both invertebrate (Gorman and Paskewitz 2001) and vertebrate innate immunity (Pham 2006; Steinhoff *et al.* 2005). Serine proteases were found to be among the genes with the greatest increases in expression in *Bd*-infected *Silurana tropicalis* (Ribas *et al.* 2009). Conversely, they also are implicated as virulence factors for parasites and pathogens, allowing them to invade or suppress host immune systems (McKerrow *et al.* 2006). Indeed, *Bd* has expanded families of both serine peptidase and metallopeptidase genes, which are purported virulence factors in the fungus (Rosenblum *et al.* 2008). Taken together, these results suggest host-protease activity and *Bd*-protease inhibition could be crucial for dealing with *Bd* infections. Clearly these classes of enzymes require further investigation, particularly in more resistant species, to more comprehensively understand *Bd* pathogenicity and host species differences in susceptibility.

### Innate defenses and interfaces of innate/adaptive immunity

We identified more than 300 genes differentially expressed between infected and control frogs that are identified as immunogenetic, indicative of extensive immune and/or inflammatory responses to *Bd* infection in *Atelopus zeteki*. These results are in stark contrast to previous transcriptome studies of other *Bd* challenged susceptible species, which showed an inconsistent or lack of robust immune response to *Bd* infections (Rosenblum *et al.* 2009, 2012).

IL genes such as (homologs of) IL-10 and IL-17 exhibited some of the greatest expression changes in infected frogs, particularly in the skin. IL-10 is produced by helper T cells, functions in B-cell maturation, and induces secretion of immunoglobulins (Rousset *et al.* 1992). It is also an anti-inflammatory cytokine and down-regulates Th2 cells and MHC class II antigens (de Vries 1995). In contrast, although also produced by helper T cells, IL-17 is proinflammatory and induces the production of many other cytokine molecules (Kolls and Lindén 2004). IL-1, -6, -8, -12, and -18 also were found to have significant increased expression in Bd-naïve frogs, all of which are linked to inflammatory responses (Akdis *et al.* 2011). However, all interleukins had either lower or nonsignificant increases in expression in frogs previously exposed to *Bd*. Strong host inflammatory responses to a pathogen can cause severe tissue damage and contribute significantly to the pathology of a disease (Graham *et al.* 2005; Sears *et al.* 2011). Our results suggest skin tissue inflammation is part of the initial response to first infection and may be an important factor in the lethality of *Bd* infection in susceptible individuals. In addition, the weaker inflammatory response in survived-infected animals could suggest either (1) hosts with inherently weaker inflammatory responses cope better with infection or (2) dampening of inflammation is an acquired response due to prior exposure. Experimental modification of inflammatory responses in highly *Bd*-susceptible species could greatly increase our understanding of levels of susceptibility to *Bd* in different species or even within populations of a single species.

The great number of cytokines identified involved in immune signaling and regulation were predominantly more highly expressed in all three tissues studied. A substantial proportion of those found in the skin (*e.g.*, CCL4, CCL19, CXCL10, CXCL14) are attractants for antigen-presenting cells such as dendritic cells and macrophages, which are thought to be key to initiating downstream acquired immune responses to *Bd* (Richmond *et al.* 2009). Also of interest, CCL27 is associated with homing of memory T lymphocytes to the skin and plays a role in T-cell−mediated inflammation of the skin (Homey *et al.* 2002). Genes of the classical complement pathway were also found to be more expressed in all tissues both infected treatment groups. In contrast to our results, previous studies have detected decreased expression of components of the classical complement pathway (Rosenblum *et al.* 2009, 2012).

In theory, toll-like receptors (TLRs) should play a key role in linking early innate responses to *Bd* to downstream acquired immunity (Richmond *et al.* 2009) given their ability to recognize a variety of other fungal pathogens in vertebrates (Luther and Ebel 2006; Roeder *et al.* 2004). These evolutionarily conserved receptors recognize chemical signatures in a wide variety of microorganisms, including fungi, and are expressed by a number of cells involved in both innate and acquired immunity (*e.g.*, T and B cells, macrophages, and dendritic cells). However, previous studies in other susceptible species found no evidence for TLR activation during infection (Rosenblum *et al.* 2009, 2012). Infected *A. zeteki* exhibited increased expression in seven TLR genes, such as TLR2 and TLR5, in at least one of the three tissues studied. Importantly TLR2, expressed by a variety of leukocytes, is known (at least in mammals) to recognize fungal pathogens and mediates pro-inflammatory responses (Goodridge and Underhill 2008). Curiously, TLR5 had the greatest increase in expression in all tissues, particularly in the skin of naïve-infected frogs. TLR5 is the only protein-binding receptor highly conserved across vertebrates from fish to mammals (Yoon *et al.* 2012) and only recognizes the protein flagellin of flagellated bacteria (Hayashi *et al.* 2001). This result indicates that naïve-infected animals were possibly responding to increased secondary bacterial infections during the course of *Bd* infection. Secondary bacterial infections have been linked to mortality during fungal infections in amphibians (Taylor *et al.* 1999). However, the possibility that this contributes to mortality in *Bd*-infected amphibians remains untested.

A great deal of work on innate amphibian resistance to *Bd* has focused on skin AMPs (Conlon 2011b; Voyles *et al.* 2011). However, we found no such sequences within our *de novo* assembly of *A. zeteki*. Previous studies have failed to characterize AMPs in other experiments using toads (Family Bufonidae) (Conlon 2011a) and in other species AMPs are only expressed at an early stage of infection (Ribas *et al.* 2009); therefore, our results corroborate that significant variation exists among species in the expression and use of AMPs against *Bd* infection.

Perhaps the most striking gene expression increase in infected individuals was the chitinase CHIT1, a key enzyme for the degradation of fungal cell walls. Increased expression of this gene was found in both infection groups compared to controls. However, CHIT1 expression was significantly greater in the spleens of survived-infected than naïve-infected frogs (with a similar but nonsignificant trend in the skin). In fact, CHIT1 exhibited one of the greatest expression changes of all genes in the spleen of survived-infected individuals. The greater expression levels observed in frogs previously exposed to *Bd* suggests some form of acquired or primed response in the expression of chitinase. Moreover, *in vitro* studies by Fites *et al.* (2013) demonstrate the immunosuppressive activity of *Bd* may be cell wall derived. Their results, coupled with our findings, indicate there may be important coevolutionary dynamics between *Bd* cytotoxicity and amphibian host chitinase activity that warrant further investigation.

### Significant acquired immune gene expression in response to *Bd* infection

The ability of amphibians to develop specific acquired immune responses to *Bd* is still debated (Richmond *et al.* 2009; Voyles *et al.* 2011). On the one hand, certain species increase antibody production after being inoculated with heat-killed *Bd* (Ramsey *et al.* 2010), frogs show increased survival time upon re-infection after previous exposure (Murphy *et al.* 2011), and specific MHC class II alleles can confer increased survival (Savage and Zamudio 2011). In contrast, immunization trials have been generally unsuccessful (Rollins-Smith *et al.* 2009; Stice and Briggs 2010), and in transcriptional studies in other susceptible species investigators found no or very few changes in expression of genes related to acquired immunity in other species (Rosenblum *et al.* 2009, 2012). In this study, we found a suite of genes with increased expression in infected *A. zeteki* directly related to acquired immune responses. Genes encoding B-cell−specific proteins and receptors, T-cell markers, and T-cell receptor components were all found to have increased expression in the skin and intestine of both infected groups. In addition, both class I and II MHC genes showed significantly increased expression in both naïve-infected and survived-infected skin samples compared with uninfected controls. Taken together, these findings indicate substantial activation of acquired immune pathways in the skin of *Bd*-infected frogs. Moreover, increased immunoglobulin expression was observed in all three tissues of infected frogs. In the skin, some immunoglobulin genes exhibited significant elevation in survived-infected frogs compared with uninfected individuals but not in naïve-infected *vs.* uninfected comparisons, suggestive of a memory response at the site of infection in individuals that were previously *Bd* exposed.

### Species differences in immune responses

Previous transcriptome studies of responses to *Bd* infection in three frog species (*Silurana tropicalis*, *Rana mucosa*, and *Rana sierrae*) were consistent with our findings that *Bd* appears to influence skin integrity and water/ion balance (Rosenblum *et al.* 2009, 2012). However, both studies propose there is an inconsistent or lack of robust immune response in these susceptible species. Our results clearly contradict these findings; we found significant increases in expression of all major immune pathways in a species highly susceptible to chytridiomycosis. Moreover, specific differences included increased expression of TLR genes (none found previously), complement genes (decreased in prior studies), and many acquired immune genes (none or few found in other species). Although it is possible that other susceptible species have weak immune responses to *Bd*, it is also possible that these contrasting findings are due to the greater sensitivity of RNA-Seq gene expression analyses over microarray-based experiments used in prior studies (Marioni *et al.* 2008; Wang *et al.* 2009). In addition, contrasting immune responses to *Bd* may result from: (1) underlying interspecific host differences in immune gene expression (Whitehead and Crawford 2006), (2) different *Bd* strains used in experiments (Berger *et al.* 2005b; Fisher *et al.* 2009a), (3) different experimental temperatures (Andre *et al.* 2008; Stevenson *et al.* 2013), (4) different numbers of zoospores inoculated (>1,000,000 *vs.* 30,000), and (5) timing of collection of tissue samples (72-hr or 10−21 d in *S. tropicalis* and 3 or 16 d in *Rana* sp.). Further work to characterize gene expression within species across a range of conditions (*e.g.*, temperature, *Bd* strain, zoospore dose) and across multiple species using controlled experimental conditions will allow us to fully elucidate the mechanisms underpinning species differences in immune response to *Bd* infection. In addition, important gene expression differences found in such studies will also warrant validation via qPCR, which remains the “gold-standard” method for measuring gene expression.

### *In vivo* indicators of immune suppression by *Batrachochytrium dendrobatidis*

Although *Bd* clearly elicits an extensive immunological response at the transcriptional level in *A. zeteki*, this species is still highly susceptible. In our experiment, all infected individuals, even those that previously survived *Bd* infection, succumbed to chytridiomycosis (*i.e.*, clinical signs of disease progressed to loss of righting reflex). This finding suggests that these substantial immunogenetic changes are insufficient to survive infection by the *Bd*.

A potential mechanism for the failure of immune responses to clear infection could be that *Bd* elicits the “wrong” immune response, *i.e.*, immune or inflammatory pathways that are activated are ineffective against the chytrid pathogen, as is seen in some chronic infections (Ferrero 1997; Zajac *et al.* 1998). Alternatively, susceptibility may be simply a problem of magnitude; frogs are mounting immune responses, but at a level insufficient to tackle infections. Finally, the time to death by *Bd* in this susceptible species could be insufficient for mounting a substantial antibody response; however, experimentally infected individuals survived 22−33 d postinoculation and antibody production in frogs is typically at a maximum around 14 days under optimal conditions (Du Pasquier *et al.* 1989; Maniero *et al.* 2006; Marchalonis 1971; Ramsey *et al.* 2010). In addition, resistant individuals begin to clear *Bd* infection after 14−30 days (Ramsey *et al.* 2010; Savage and Zamudio 2011); therefore, insufficient time to mount an antibody defense seems an unlikely explanation for host susceptibility in this case. However, as antibody response times are uncharacterized in this species, the possibility cannot be definitively ruled out with our data.

A final plausible explanation is that *Bd* is capable of evading or suppressing immune functions in susceptible species (Ribas *et al.* 2009; Rollins-Smith *et al.* 2011; Rosenblum *et al.* 2008, 2009). Fites *et al.* (2013) provided the first evidence for this hypothesis, demonstrating *Bd* inhibition of host lymphocyte proliferation. We found B-cell−related genes, T-cell markers, and many T-cell receptor components (including alpha, beta, delta, and gamma chains) have lower expression in infected frogs than uninfected individuals. Given that tissues were sampled in the late stages of infection, this could be a result of cell migration/localization to the skin. However, we found nowhere near as comprehensive a set of genes with increased expression in infected skin samples. Alternatively, the strong proinflammatory responses observed may actually be dampening lymphocyte production as is seen during both acute and chronic inflammation in mammals (Gabrilovich and Nagaraj 2009; Hotchkiss *et al.* 2009).

Decreased gene expression alone cannot discern between pathogen- and host-mediated down-regulation of lymphocytes; however, these results correlate with recent findings *in vitro* that show *Bd* inhibits splenic lymphocyte proliferation (Fites *et al.* 2013). Our data are the first *in vivo* indication that *Bd* may actually be suppressing B- and T-cell production, differentiation, and/or maturation in the spleen. Although not directly quantified, we also observed that spleen volume in infected frogs was reduced compared with uninfected individuals, a condition that is indicative of immune suppression, because spleen volume is generally increased in vertebrates responding to infections (Brown and Brown 2002; Vicente *et al.* 2007). Fites *et al.* (2013) also demonstrated *Bd* induces apoptosis, which could account for our qualitative observation.

Mechanisms of fungal immunosuppression include the exploitation of host immune-related receptors (Brandhorst *et al.* 2004) and the release of toxins (Bondy and Pestka 2000). *Bd* may produce toxic factors (McMahon *et al.* 2013) that inhibit immune responses *in vitro*, and these factors may be derived from the fungal cell wall (Fites *et al.* 2013). Although these cannot be tested with our data, both warrant further *in vivo* investigation in terms of *Bd* activity. In other cases, it seems fungi can elicit ineffective host responses to infection, which can actually lead to immunosuppression. For example, *Paracoccidioides brasiliensis* attracts regulatory T cells (T_regs_) to sites of infection, leading to down-modulation of effector immune responses and the long-term presence of the fungus in granulomas (Moreira *et al.* 2008). Migration of T_regs_ is commonly associated with chemokine receptor 4 (CCR4) and T_reg_ infiltration into secondary lymph tissues, via CCL19, leads to suppression of both B and T cells (Mailloux and Young 2010). In addition, T_regs_ often express IL-10 at sites of infection/inflammation, which can minimize host tissue damage but also allows parasites or viruses to evade effector responses and proliferate (Belkaid *et al.* 2006). In our study CCR4, CCL19, and IL-10 all had significantly increased expression in infected frogs. Taken together, with the observed decreases in expression of T- and B-cell−specific genes in the spleen, the possibility that *Bd* activation of T_reg_, may contribute to immune suppression is a promising line of inquiry for our understanding of the outcome of chytridiomycosis in hosts with differing susceptibility.

Despite the high susceptibility of *Atelopus zeteki* to chytridiomycosis, we found wide-ranging and robust immune responses to experimental *Bd* infections. Our study provides a detailed overview of immunogenetic changes in response to the fungal pathogen including increased expression of many innate immune and inflammatory pathways and adaptive immune genes such as immunoglobulins and MHC. Moreover, genes thought to be essential to antifungal activity, such as chitinase and serine-type proteases, exhibited greater expression in animals previously exposed to *Bd*, indicating that this species may mount acquired immune responses to *Bd*.

We propose that susceptibility to chytridiomycosis in this case is not necessarily due to lack of acquired immune responses, but instead is caused by some failure in the efficacy of the responses. Although ineffective immune pathway activation or insufficient levels of immune effectors cannot be ruled out, the reduced expression levels of B- and T-cell genes in the spleen of infected animals compared with controls suggest immunosuppression by the pathogen itself, reinforcing recent findings of *in vitro* studies. Our results provide important in-depth transcriptomic resources for future studies of *Bd* susceptibility and anti-*Bd* amphibian immune mechanisms. In addition, we highlight the need for transcriptomic studies on highly resistant frog species to further disentangle potential mechanisms of chytridiomycosis susceptibility.

## Supplementary Material

Supporting Information
