# Supplementary material for: Fighting a Losing Battle: Vigorous Immune Response Countered by Pathogen Suppression of Host Defenses in the Chytridiomycosis-Susceptible Frog Atelopus zeteki
Source: G3 (Bethesda). 2014 May 19;4(7):1275–89. doi: 10.1534/g3.114.010744 (PMC4455776; doi:10.1534/g3.114.010744)
Supplement: Supporting Information [file supp_4_7_1275__index.html]

Fighting a Losing Battle: Vigorous Immune Response Countered by Pathogen Suppression of Host Defenses in the Chytridiomycosis-Susceptible Frog Atelopus zeteki — Supporting Information 

# Fighting a Losing Battle: Vigorous Immune Response Countered by Pathogen Suppression of Host Defenses in the Chytridiomycosis-Susceptible Frog *Atelopus zeteki*

## Supporting Information for Ellison *et al.*, 2014

**Files in this Data Supplement:**

- Table S1 - Over-represented GO terms in gene expression clusters. (.xls, 131 KB)
- Table S2 - Complete list of all genes related to immune system function found to be differentially expressed among treatment groups in the skin of *Atelopus zeteki*. (.xls, 4 MB)
- Table S3 - Complete list of all genes related to immune system function found to be differentially expressed among treatment groups in the spleen of *Atelopus zeteki*. (.xls, 2 MB)
- Table S4 - Complete list of all genes related to immune system function found to be differentially expressed among treatment groups in the intestine of *Atelopus zeteki*. (.xls, 4 MB)
